# Clinical application of serum biomarkers for detecting and monitoring of chronic plaque psoriasis

**DOI:** 10.3389/fmolb.2023.1196323

**Published:** 2023-07-21

**Authors:** Criselda Jean G. Cruz, Chao-Chun Yang

**Affiliations:** ^1^ Department of Dermatology, National Cheng Kung University Hospital, College of Medicine, National Cheng Kung University, Tainan, Taiwan; ^2^ International Center for Wound Repair and Regeneration, National Cheng Kung University, Tainan, Taiwan

**Keywords:** psoriasis, ELISA, biomarkers, biologics, interleukins, skin inflammation

## Abstract

Psoriasis, a chronic, multisystemic inflammatory disease affecting millions of people globally, manifests as erythematous, thick, scaly plaques on the skin. Clinical evaluation remains to be the benchmark for diagnosis and monitoring of this debilitating disease. With current advancements in targeted molecular therapy for psoriasis such as biologics, molecular detection methods may also help guide clinical decisions and therapeutic strategies through quantification of circulating biomarkers, which could reflect the underlying pathogenic events happening at a certain point of the disease course. In this review, we will discuss how biomarkers are detected in serum samples using enzyme-linked immunosorbent assay (ELISA). This review will feature candidate biomarkers supported by clinical data for psoriasis including, but not limited to, cytokines, chemokines, adipokines, and antimicrobial peptides. A better understanding of the common method used for biomarker detection would enable physicians to interpret and correlate laboratory results with the disease pathogenesis and clinical outcomes, e.g., severity assessment and/or therapeutic response. With better health outcomes as the main goal, the utility of such information to evaluate and even predict treatment response would be a major step closer towards patient-tailored management.

## 1 Introduction

Psoriasis is a chronic, immune-mediated condition manifesting as thick, raised, scaly plaques (World Health Organization, 2016). Psoriasis vulgaris or chronic plaque psoriasis constitutes 90% of all diagnosed psoriasis cases, some presenting with other systemic comorbidities, e.g., psoriatic arthritis, cardiovascular diseases, diabetes mellitus, cancer, and depression, thereby causing serious debilitation in one’s quality of life (Di Meglio et al., 2014; World Health Organization, 2016; Rendon and Schäkel, 2019).

Onset and exacerbation of psoriasis is multifactorial, including genetic and environmental factors (Armstrong and Read, 2020). Its complex inflammatory processes involve members of the adaptive immune system. Excessive activation of myeloid dendritic cells occurs due to the cytokines secreted by plasmacytoid dendritic cells (pDC), keratinocytes, macrophages, and T cells, resulting in the secretion of interleukin (IL)-12 and IL-23 (Alwan and Nestle, 2015). IL-12 drives naive T cells to differentiate into T_h_1 cells, which secretes interferon-gamma (IFN-γ) and tumor necrosis factor-alpha (TNF-α). IL-23 plays a critical role for T_h_17- and T_h_22-mediated cytokine release, i.e., T_h_17 secretes IL-17, IL-22, and TNF-α, while T_h_22 produces IL-22 (Armstrong and Read, 2020). Overall, these inflammatory signals lead to the skin and systemic manifestations in psoriasis, e.g., keratinocyte proliferation, immune cells infiltrating skin lesions, vasodilation, and angiogenesis (Nestle et al., 2009; Armstrong and Read, 2020).

Skin biopsy is an invasive procedure seldom used in diagnosing psoriasis (Greaves and Weinstein, 1995). Less invasive diagnostics offer the advantage of clinching the diagnosis while allowing repetitive measurements when warranted. Circulating proteins, also referred to as biomarkers, can be quantified through less invasive procedures and can indicate disease activity and progression, i.e., significant deviation from normal levels likely reflects an abnormal process occurring within the body (Molteni and Reali, 2012; Aronson and Ferner, 2017).

**FIGURE 1:**
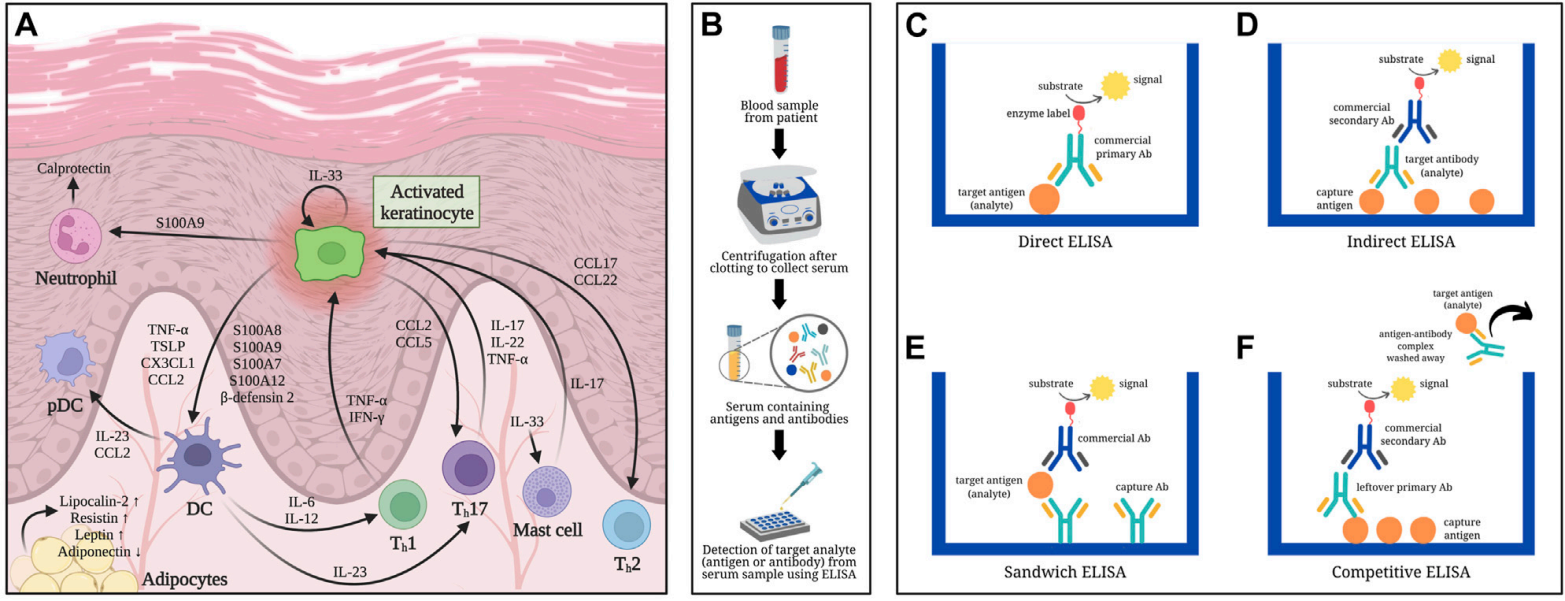
Overview of psoriasis-related serum biomarkers determined using ELISA-based methods: **(A)** Involvement of serum biomarkers in the pathogenesis of psoriasis. **(B)** Schematic diagram of serum collection from blood samples: Whole blood from a patient is allowed to clot first then centrifuged to separate the serum from clotted blood cells. **(C)** Direct ELISA: Antigens (analyte) from serum are immobilized onto a polystyrene microtiter plate followed by a blocking step (i.e., using bovine serum albumin) to cover any remaining plastic surface prior to adding the commercial enzyme-linked antibody (Ab) that would specifically bind to the antigens being measured. Signal emitted is directly proportional to the quantity of analytes. **(D)** Indirect ELISA: The microtiter plate is first coated with capture antigens, which would bind the antibodies (analyte) referred to as primary antibody from the serum sample. An enzyme-labeled secondary antibody (usually commercially obtained), which is host-specific to the primary antibody, is then added to detect the analytes captured onto the microtiter plate, hence emitting a signal proportional to the amount of analyte. **(E)** Sandwich ELISA: Capture antibodies specific to the analyte (antigen) are immobilized onto the wells of a microtiter plate, followed by the addition of the serum sample containing the antigens of interest. Once the antigens bind to the capture antibodies, a second antibody specific to a different epitope region of the analyte is added into the wells. The second antibody may or may not be labeled; in the latter case, an enzyme-conjugated third antibody, which is specific to the constant (Fc) region of the second antibody, would be included and act as the reporting antibody. The signal detected is directly correlated to the amount of antigens detected in the sample. **(F)** Competitive ELISA: Antigens are initially pre-coated onto the wells of a microtiter plate. The sample containing antigens to be measured are preincubated with primary antibodies to create mobile antibody-antigen complexes before adding the sample mixture onto the antigen-coated microtiter plates. Antigens, when present in high amounts, would result in fewer unbound primary antibodies. After washing off the sample mixture containing the mobile antibody-antigen complexes, any free primary antibodies would be captured by the immobilized antigens in the microtiter plate. A secondary antibody against the Fc region of the primary antibody is then added to report how much of it remained in the microtiter plate. Low signal detected in competitive ELISA implies that high amount of antigens were present in the sample, resulting in less unbound primary antibodies that could bind with the capture antigens on the microtiter plate.

Biomarker studies for psoriasis aim to seek for reliable yet minimally invasive indicators of disease status and/or treatment response. Although psoriasis is often clinically diagnosed, biomarkers may serve as an aid for clinicians in quantitatively monitoring disease activity and therapeutic response. In this review, we discussed serum protein biomarkers that have been clinically correlated with psoriasis, as summarized in Figure 1A and Table 1. Immunoassay methods were also briefly discussed in the context of serum biomarker detection. In doing so, we hope to provide clinicians with the fundamentals of immunoassays to facilitate better interpretation of immunoassay results and develop personalized and effective therapeutic strategies.

**TABLE 1:** Serum biomarkers for psoriasis in correlation with disease severity and/or treatment response.

Serum biomarker	Serum level: Psoriasis vs. healthy	Correlates with severity?^a^	Reflects treatment response?^a^	References
IL-6	up	Y	Y (adalimumab, infliximab)	Arican et al. (2005), Shibata et al. (2009), Muramatsu et al. (2017), Michalak-Stoma et al. (2022)
IL-12	up	Y	Y (etanercept)	Arican et al. (2005), Takahashi et al. (2010), Brito-Luna et al. (2016), Lu et al. (2016)
IL-17	up	Y	Y (etanercept)	Arican et al. (2005), Caproni et al. (2009), Takahashi et al. (2010), Yilmaz et al. (2012), Nassar et al. (2022)
IL-22	up	Y	Y (etanercept, guselkumab, ustekinumab)	Boniface et al. (2007), Caproni et al. (2009), Lo et al. (2010), Shimauchi et al. (2013), Dudakov et al. (2015), Gordon et al. (2019), Wawrzycki et al. (2019), Philipp et al. (2020)
IL-33	up	N		Balato et al. (2012), Mitsui et al. (2016), Li et al. (2017), Borsky et al. (2020)
IFN-γ	up	N		Jacob et al. (2003), Arican et al. (2005), Moustafa et al. (2009), Takahashi et al. (2010), Kurtovic and Halilovic (2018)
TNF-α	up	N		Arican et al. (2005), Takahashi et al. (2010), Kyriakou et al. (2014), Ovcina-Kurtovic and Kasumagic-Halilovic (2022)
TSLP	up	Y		El-Ghareeb et al. (2019)
HMGB1	up	N		Bergmann et al. (2016), Strohbuecker et al. (2019), Borsky et al. (2020)
IL-10	no difference			Dowlatshahi et al. (2013), Bai et al. (2018)
CX3CL1	up	Y		Congjun et al. (2015)
TARC/CCL17	up	N		Purzycka-Bohdan et al. (2022)
CCL2	up	N		Purzycka-Bohdan et al. (2022)
CCL3	up	N		Purzycka-Bohdan et al. (2022)
CCL5	up	N		Purzycka-Bohdan et al. (2022)
CCL18	up	N		Purzycka-Bohdan et al. (2022)
CCL22	up	N		Purzycka-Bohdan et al. (2022)
Lipocalin-2	up (acute types)	Y		Nguyen and Nguyen (2022)
Resistin	up	Y		Bai et al. (2018), Seth et al. (2020)
Adiponectin	down	N		Shibata et al. (2009), Oh et al. (2014)
Leptin	up	Y		Cerman et al. (2008), Zhu et al. (2013), Oh et al. (2014)
Calprotectin	up	Y	Y (methotrexate)	Hamza et al. (2019), Scali et al. (2022), Li et al. (2023)
S100A8	up	Y		Benoit et al. (2006), Matsunaga et al. (2021)
S100A9	up	Y		Benoit et al. (2006), Matsunaga et al. (2021)
S100A7	up	Y		Wilsmann-Theis et al. (2016)
S100A12	up	Y		Wilsmann-Theis et al. (2016)
β-defensin 2	up	Y	Y (secukinumab, tofacitinib)	Jin et al. (2017), Morita et al. (2020)
FGL1	down	Y		Sun et al. (2022)
OAS2	up	Y		Zhou et al. (2020)

^a^ Y: yes, N: no.

## 2 Principles of protein-based immunoassay for serum biomarker detection

Biomarker detection may require a few drops of blood by finger prick method to a few milliliters by venipuncture (Paul and Veenstra, 2022). Serum can be separated from other blood components by centrifugation after allowing blood to coagulate (Figure 1B). Antibody-based techniques, e.g., enzyme-linked immunosorbent assay (ELISA), have high sensitivity and specificity in detecting proteins from serum. ELISA utilizes the specific binding between proteins and antibodies in producing a measurable signal (Alhajj and Farhana, 2023).

Regarded as the gold standard for immunoassays, ELISA was first described in 1972 as a simple yet sensitive analytical technique for antibody quantification, detecting less than 1 ng/mL of antibody (Engvall and Perlmann, 1972). The quantifiable signal results from an enzyme-substrate reaction indicating that the analyte is present and binds the reporting antibody conjugated with the enzyme (Figures 1C–F). Different types of ELISA (direct, indirect, sandwich, and competitive) mainly vary in the number of steps involved.

Direct ELISA is a straightforward manner of immunoassay (Figure 1C), wherein the antigens (analyte) from serum acts as capture molecules for its specific reporting enzyme-linked antibody (Aydin, 2015; Alhajj and Farhana, 2023). Indirect ELISA enables detection of antibody from serum when captured by immobilized antigens followed by the addition of an enzyme-labeled secondary antibody (Figure 1D) (Aydin, 2015; Alhajj and Farhana, 2023). Sandwich ELISA, as the name implies, involves two antibodies that capture the antigen of interest in between (Figure 1E). Commonly used for biomarker detection, sandwich ELISA has specific primary antibodies immobilized instead of directly coating the microtiter plate with analytes, thereby increasing its specificity while reducing nonspecific binding (Aydin, 2015; Alhajj and Farhana, 2023). Competitive ELISA is another platform that allows detection of antigens or biomarkers (Figure 1F). It is often useful when the analyte is small and difficult to be “sandwiched” in between antibodies (Gan and Patel, 2013; Aydin, 2015; Alhajj and Farhana, 2023).

Other techniques for biomarker detection include mass spectrometry, flow cytometry, and protein microarrays; however, these are not readily accessible in clinical settings hence were excluded in this review. ELISA is a well-established method that is feasible in most laboratories as commercial kits are often readily available. Given its high sensitivity and specificity, sandwich ELISA is most commonly used in biomarker assays (Del Campo et al., 2015). The subsequent section discusses psoriasis-related protein biomarkers, most of which were studied using sandwich-type ELISA.

## 3 Serum biomarkers for psoriasis

Candidate serum biomarkers for psoriasis discussed in this review include cytokines, chemokines, adipokines, and antimicrobial peptides, as summarized in Table 1.

### 3.1 Cytokines and chemokines

During inflammation, cytokines and chemokines are secreted by various immune cells to mediate signaling pathways and recruit effector cells towards the affected sites, hence may serve as candidate biomarkers for long-term inflammatory diseases such as psoriasis.

#### 3.1.1 IL-6

With its implications on epidermal and dermal growth and differentiation, IL-6 is being explored as a dendritic cell (DC)-produced inflammatory marker as it induces T cell conversion to T_h_17, which in effect produces other inflammatory cytokines (Goodman et al., 2009; Pietrzak et al., 2020; Kutsuna et al., 2022). Baseline IL-6 levels in serum of psoriasis patients were found to be significantly elevated compared to healthy individuals (Arican et al., 2005; Shibata et al., 2009; Michalak-Stoma et al., 2022). Moreover, a significant correlation was noted between serum IL-6 and PASI scores in psoriasis vulgaris patients (Muramatsu et al., 2017). Therapeutic response was also reflected by significant decrease in serum IL-6 levels after treatment with biologics such as adalimumab and infliximab.

#### 3.1.2 IL-12

IL-12 plays a role in T-cell-mediated immunity and is considered as one of the key cytokines responsible for formation and persistence of psoriatic plaques (Ergen and Yusuf, 2018). Patients with plaque psoriasis exhibited elevated serum IL-12, which also correlates with PASI scores (Arican et al., 2005; Takahashi et al., 2010; Brito-Luna et al., 2016). In addition, patients who were classified as non-responders to a 24-week treatment of etanercept were noted to have lower baseline IL-12 serum levels than the responders, hence may be suggestive of IL-12 as a potential biomarker or predictor for clinical response to etanercept, which is a TNF-inhibitor used to treat psoriasis (Lu et al., 2016). Moreover, serum IL-12 level could potentially serve as an indicator for clinical response to etanercept with 50% sensitivity and 96% specificity. In this study, clinical response was defined by a reduction of 75% in the PASI score after treatment.

#### 3.1.3 IL-17

Produced by various immune cells such as T_h_17, neutrophils, and mast cells, IL-17 is a key cytokine responsible for keratinocyte proliferation and production of other cytokines and antimicrobial peptides in psoriasis (Veldhoen et al., 2006; Lin et al., 2011; Keijsers et al., 2014; Dyring-Andersen et al., 2017). Serum IL-17 concentration was higher in individuals with chronic plaque psoriasis than in healthy controls (Takahashi et al., 2010; Nassar et al., 2022). IL-17 in serum also correlated with severity in psoriasis patients (Arican et al., 2005; Takahashi et al., 2010; Yilmaz et al., 2012). It has been reported that IL-17 levels in serum decreased after treatment with etanercept, suggesting that IL-17 is a potential biomarker for both disease severity and therapeutic response monitoring (Caproni et al., 2009).

#### 3.1.4 IL-22

Described as a proinflammatory cytokine produced by CD4 memory T cells (e.g., T_h_1, T_h_17, T_h_22), IL-22, a member of the IL-10 family, have been found to be significantly elevated in patients with psoriasis compared to healthy controls (Boniface et al., 2007; Lo et al., 2010; Shimauchi et al., 2013; Dudakov et al., 2015; Gordon et al., 2019; Wawrzycki et al., 2019). These studies also demonstrated that IL-22 levels exhibit a positive correlation with disease severity based on PASI scores, making it a potential cytokine suitable for monitoring psoriasis activity. IL-22 may also be a biomarker indicating treatment response due to the notable decrease in serum IL-22 levels after treatment with either etanercept, guselkumab (anti-IL-23) or ustekinumab (anti-IL-12/23) (Caproni et al., 2009; Gordon et al., 2019; Philipp et al., 2020).

#### 3.1.5 IL-33

Regarded as both cytokine and damage-associated molecular pattern, IL-33 is released upon keratinocyte damage in psoriasis then activates inflammatory pathways in an autocrine manner (De la Fuente et al., 2015; Zeng et al., 2021). IL-33 secreted by endothelial cells could activate mast cells, which then initiate inflammation in unaffected psoriatic skin (Theoharides et al., 2010). Studies have reported that IL-33 levels are elevated in the serum of psoriasis patients than the healthy controls, although no correlation found between IL-33 levels and PASI scores (Mitsui et al., 2016; Li et al., 2017; Borsky et al., 2020). Despite having reported to be increased in other skin conditions such as vitiligo and atopic dermatitis (Tamagawa-Mineoka et al., 2014; Vaccaro et al., 2016), IL-33 elevation was significantly higher in psoriatic than in AD lesions, hence lesional IL-33 seem to correlate strongly with psoriasis in a localized manner (Balato et al., 2012).

#### 3.1.6 IFN-γ

IFN-γ is a T_h_1-derived cytokine that mediates downstream processes favoring keratinocyte proliferation, a key occurrence in psoriasis pathogenesis (Nickoloff and Nestle, 2004). Although there was no overall significant correlation to disease type or severity in terms of PASI score, elevated IFN-γ serum concentration has been associated with active psoriasis in multiple studies (Jacob et al., 2003; Arican et al., 2005; Moustafa et al., 2009; Takahashi et al., 2010; Kurtovic and Halilovic, 2018). Altogether, increased IFN-γ likely indicates presence of psoriasis, though might not be suitable in terms of assessing disease severity due to conflicting reports regarding its correlation with PASI scores.

#### 3.1.7 TNF-α

TNF-α is another key proinflammatory cytokine that promotes immune cell migration towards the skin resulting in keratinocyte proliferation, which is one of the hallmark manifestations of psoriasis (Conrad and Gilliet, 2018; Ogawa et al., 2018; Ovcina-Kurtovic and Kasumagic-Halilovic, 2022). In psoriasis, affected keratinocytes produce excessive TNF-α, hence activating dendritic cells to produce IL-12 and IL-23. These cytokines promote T cell differentiation into T_h_1 and T_h_17, which produce more TNF-α and other proinflammatory cytokines such as IL-17 and IFN-γ, leading to the pathologic manifestations of psoriasis, i.e., keratinocyte hyperproliferation, acanthosis, parakeratosis, and hypogranulosis (Conrad and Gilliet, 2018; Ogawa et al., 2018). Serum TNF-α was found to be significantly higher in those with psoriasis than those without (Arican et al., 2005; Takahashi et al., 2010; Kyriakou et al., 2014; Ovcina-Kurtovic and Kasumagic-Halilovic, 2022); however, majority of these studies report that its correlation with PASI scores was not significant.

#### 3.1.8 Thymic stromal lymphopoietin (TSLP)

TSLP is a proallergic keratinocyte-derived cytokine, which has been described to induce dendritic cell maturation and IL-23 production in psoriasis pathogenesis (Volpe et al., 2014). Serum TSLP has been reported to be elevated in psoriasis patients with a significant correlation to disease severity (El-Ghareeb et al., 2019). TSLP may be a potential indicator for early detection and diagnosis of active psoriasis.

#### 3.1.9 High-mobility group box 1 (HMGB1)

Previous studies found that HMGB1, a proinflammatory cytokine, was significantly increased in sera of psoriasis patients compared to healthy controls (Bergmann et al., 2016; Strohbuecker et al., 2019; Borsky et al., 2020); however, its relationship with disease severity is yet to be established.

#### 3.1.10 IL-10

Despite being an anti-inflammatory cytokine expected to be downregulated in the presence of psoriasis, no significant difference has been noted in the baseline serum IL-10 levels between patients with psoriasis and healthy individuals (Dowlatshahi et al., 2013; Bai et al., 2018). In attempt to quantify serum IL-10 concentrations in psoriasis patients, multiple cytokine analysis approach was applied by Michalak-Stoma et al. (2022); however, signal intensities for IL-10 were insufficient for quantification, implying that this anti-inflammatory cytokine may not be an ideal serum biomarker for psoriasis.

#### 3.1.11 Fractalkine (CX3CL1)

Fractalkine, a membrane-bound chemokine, was significantly elevated in the serum of psoriasis patients compared to healthy individuals (Congjun et al., 2015). In the same study, the positive correlation between CX3CL1 serum levels and PASI scores was also demonstrated, suggesting the potential utility of this biomarker to assess disease severity.

#### 3.1.12 Thymus and activation-regulated chemokine (TARC/CCL17) and other chemokines

Thymus and activation-regulated chemokine or CCL17 serum levels were significantly higher in patients with chronic plaque psoriasis compared to that of the healthy controls (Purzycka-Bohdan et al., 2022). Moreover, serum TARC correlated positively with pruritus assessed using the visual analog scale, but no overall significant correlation was seen between serum TARC and PASI scores. Other chemokines, namely, CCL2/MCP-1, CCL3/MIP-1α, CCL5/RANTES, CCL18/PARC and CCL22/MDC, although not significantly correlated with disease severity, were also found to be elevated in patients with chronic plaque psoriasis.

### 3.2 Adipokines

Adipose-tissue derived mediators called adipokines have been linked with metabolic disturbances and chronic inflammation including psoriasis. Immune dysregulation in psoriasis leads to secretion of adipokines into the bloodstream, hence such molecules potentially reflect disease activity (Słuczanowska-Głabowska et al., 2023).

#### 3.2.1 Lipocalin-2

Detected in blood samples through ELISA, lipocalin-2 is an adipokine that has been correlated with disease severity in psoriasis patients. In a study of 62 patients with psoriasis, the BSA and PASI scores in cases of psoriatic erythroderma and psoriasis vulgaris, as well as the severity score of generalized pustular psoriasis, were all noted to have positive correlation with lipocalin-2 levels (Nguyen and Nguyen, 2022). Furthermore, lipocalin-2 levels were significantly higher in patients with acute types of psoriasis than in chronic types, indicating the potential of this adipokine as a biomarker for psoriasis accompanied with acute inflammation.

#### 3.2.2 Resistin

Resistin is a pro-inflammatory adipokine involved in TNF-α-related pathways in psoriasis (Nakajima et al., 2013; Müge et al., 2019). Serum resistin concentrations were significantly higher in psoriasis patients than that of healthy individuals (Bai et al., 2018). A correlation between serum resistin and PASI score was also reported, in which more severe cases of psoriasis had higher resistin levels than those with lower PASI scores (Seth et al., 2020).

#### 3.2.3 Adiponectin

Psoriasis patients exhibited lower serum adiponectin levels relative to the healthy controls (Shibata et al., 2009; Oh et al., 2014), indicating that adiponectin’s supposedly anti-inflammatory role is downregulated in psoriasis. Furthermore, no significant correlation was noted between adiponectin levels in serum of patients and their PASI scores.

#### 3.2.4 Leptin

Leptin, an energy-regulating secretory hormone from adipose tissue, has been associated with proinflammatory cytokine induction (Cerman et al., 2008). Serum leptin levels were found to be elevated in psoriasis patients compared to healthy controls and positively correlates with PASI scores (Cerman et al., 2008; Zhu et al., 2013; Oh et al., 2014).

### 3.3 Antimicrobial peptides

#### 3.3.1 S100 proteins

Calprotectin is an antimicrobial peptide and calcium-binding soluble protein secreted by monocytes and neutrophils during inflammation (Voganatsi et al., 2001). Regarded as a potential novel biomarker for psoriasis, serum calprotectin was significantly higher and correlates positively with PASI scores in psoriatic patients than healthy controls (Scali et al., 2022; Li et al., 2023). Both PASI scores and serum calprotectin levels significantly decreased after 3-month methotrexate therapy of patients with psoriasis vulgaris, demonstrating its potential as a prognostic marker for treatment response (Hamza et al., 2019). Furthermore, calprotectin might indicate disease relapse as serum levels were noted to be significantly higher in relapsed cases compared to nonrelapsed cases of psoriasis.

Calcium-binding S100 proteins A8 (S100A8) and A9 (S100A9) levels were significantly increased in both serum and stratum corneum of psoriasis patients while also positively correlating with PASI scores (Benoit et al., 2006; Matsunaga et al., 2021), demonstrating the capability of these proteins in reflecting disease severity. Additionally, serum S100A7 (psoriasin) and S100A12 (calgranulin-c) were found to be elevated in psoriasis patients, with the latter being regarded as the most promising S100 protein biomarker thus far in terms of correlating with severity of psoriasis (Wilsmann-Theis et al., 2016).

#### 3.3.2 β-defensin 2 (BD-2)

Inflammation drives the expression of BD-2 from keratinocytes (Cieślik et al., 2021). Baseline serum BD-2 levels were higher in patients with active psoriasis than healthy controls (Morita et al., 2020). Furthermore, serum BD-2 positively correlated with PASI score and was found to decrease after treatment with secukinumab, an anti-IL-17A drug. A similar trend was reported upon treatment with tofacitinib, a JAK-inhibitor (Jin et al., 2017). Serum BD-2 levels could potentially reflect psoriasis severity and treatment response to secukinumab or tofacitinib, although warranting further studies.

### 3.4 Other potential novel protein biomarkers

#### 3.4.1 Fibrinogen-like protein 1 (FGL1)

FGL1, a 68-kD protein from the fibrinogen family, was found to be significantly lower in the serum of psoriasis patients than healthy controls (Sun et al., 2022). Despite this observation, PASI scores of patients with psoriasis remained positively correlated with serum FGL1 concentration.

#### 3.4.2 2-5-Oligoadenylate synthase 2 (OAS2)

OAS2 is a potential novel biomarker identified from proteomic profiles of psoriasis patients, who exhibited significantly higher OAS2 serum levels than healthy controls (Zhou et al., 2020). The reported positive correlation between PASI score and OAS2 levels suggests that OAS2 potentially reflects disease severity. Moreover, OAS2 was shown to differentiate those with low PASI scores (PASI≤10) from healthy controls.

## 4 Discussion: future prospects and clinical application

Quantification of biomarkers from a small amount of blood or serum sample obtained via minimally invasive approaches offers plenty of advantages, including the willingness and increased compliance of patients, emphasizing the importance of biomarker identification and assays. One promising approach is by integrating high throughput technologies such as proteomics analysis as demonstrated in the discovery of OAS2 as a candidate biomarker for psoriasis (Zhou et al., 2020).

Establishing reliable biomarkers for psoriasis involves several stages. First is to identify detectable biomarkers that reflect key clinical outcomes (e.g., disease severity and treatment response). Validation studies, wherein biomarkers are assayed and correlated with the patients’ clinical manifestations, is preferably conducted in a larger cohort to better establish the correlation. Determining a biomarker’s correlation to the disease status (whether positive or negative), its cut-off values, sensitivity, and specificity provides useful information that can support a physician’s clinical decision-making and management of psoriasis patients.
